# Multimodal Anesthesia via Opioid-Free Analgesia and Erector Spinae Plane Block

**DOI:** 10.1155/2020/6062935

**Published:** 2020-03-27

**Authors:** Juan Carlos Luis-Navarro, Carmen Fornés-Rumbao, Ana Bella DeLaCalle-Gil, Mauricio Forero

**Affiliations:** ^1^Anesthesia-Reanimation Service, Virgen Del Rocio University Hospital, Seville, Spain; ^2^Anesthesia Department, McMaster University, Hamilton, Canada

## Abstract

Multimodal anesthesia, which combines general and epidural anesthesia, is used in surgical cases in which a large or painful incision is anticipated. However, both epidural blocks and opioid-based analgesia have limitations in application. Here, we present a case of supra-infraumbilical laparotomy in a patient whose history of neurostimulator use and marked scoliosis discouraged the placement of an epidural catheter and whose prior adverse response to opioids prohibited their use. The intraoperative and postoperative management of this patient consisted of a combination of analgesia without opioids and erector spinae plane block. Adequate analgesia was achieved, and intraoperative or postoperative opioids were not required. This case illustrates the importance of mastering alternative and multimodal analgesia techniques that can be used in place of classical analgesia techniques when classical analgesia techniques are not appropriate.

## 1. Introduction

Multimodal anesthesia, in which general and epidural anesthesia are combined, is a technique used in many cases of open abdominal surgery with wide incisions. However, performing an epidural block is not always recommended or possible in some cases. For instance, in patients with previous spinal surgery or epidural devices, epidural blocks may be impossible because of scar tissue development. In these cases, alternative methods include intravenous opiate administration and regional nerve blocks. However, opiates can have contraindications, and regional nerve block techniques may not sufficiently provide analgesia for an extensive surgery.

Here, we present the case of a patient who required a supra-infraumbilical middle laparotomy. The patient's medical history included previous scoliosis-related surgery, implanted epidural neurostimulator, and bronchospasm response after administration of morphine and tramadol. Given the patient's history and limitations, we employed a bilateral erector spine muscle plane block. This approach has been previously employed in abdominal surgery [1–3]. However, considering the extent of the surgical procedure and the history of scoliosis-related surgery, it was possible that the block would not cover the entire surgical field in this case. Thus, we complemented the block with opioid-free analgesia [4, 5], which is increasingly used in patients with a history of opioid-related, intolerable side effects and particularly in laparoscopic surgery cases. Using this approach we were able to achieve good intraoperative and postoperative analgesic control.

## 2. Case Presentation

**Figure 1:**
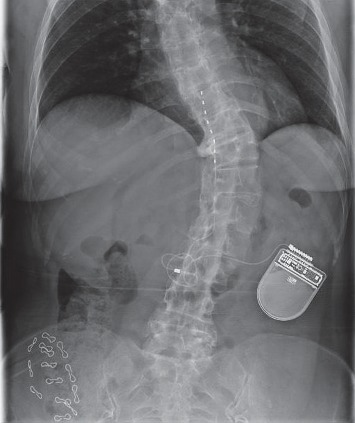
X-ray image of the thoracic and lumbar spine. The degree of scoliosis and the presence of the linear electrode of the spinal neurostimulator at low thoracic level can be seen.

**Figure 2:**
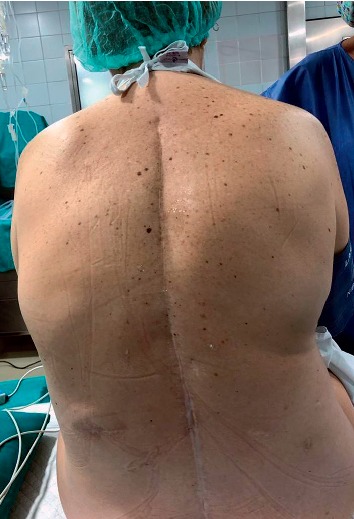
Image of the back in which the scar is seen on the spinous process in the thoracic and lumbar region, as well as the projection corresponding to the implant zone of the neurostimulator.

We treated a 52-year-old woman who was diagnosed with stage IIIC endometrial adenocarcinoma with suspected pelvic and aortocaval adenopathic involvement. The patient's relevant medical history included scoliosis and bronchospasm with severe dyspnea and diaphoresis after the administration of morphine and tramadol. Her surgical history included thoracic and lumbar surgery for scoliosis correction, followed by removal of hardware material because of failure, and the surgical implantation of a Boston Scientific spinal neurostimulator with linear electrodes at the low thoracic level (Figure 1) for postlaminectomy syndrome, which caused chronic pain and episodes of decreased strength in the lower limbs. The patient was very dependent on the neurostimulator, which was already turned off in the operating room. Because of these interventions, the patient had a scar over the spinous process from the C7 to L5 process (Figure 2). Planned intervention consisted of a supra and infraumbilical middle laparotomy for hysterectomy, right adnexectomy, pelvic and aortocaval lymphadenectomy, and sigmoid resection and colostomy.

Given the patient's history, we decided on a combined approach to intraoperative analgesia using a bilateral erector spinae plane block (ESP block) and the placement of catheters for postoperative pain control, together with an opioid-free analgesic (OFA) technique. We chose this approach because the invasiveness of the surgical procedure and the patient's history of scoliosis surgery raised the possibility that a bilateral ESP block would not cover the entire surgical field. The patient gave informed consent for the anesthetic plan as well as consent for the publication of images.

The ESP block was performed with the patient in sitting position using a modified version of the technique described by Forero [1]. Identification of the puncture level was not possible by palpation of the spinous processes alone because of scarring from previous scoliosis surgeries. Thus, a high-frequency ultrasound probe (LA523 4–13 MHz by Esaote MyLabFive®) covered with a sterile sheath was used to identify the lower edge of the right rhomboid muscle, which corresponds to the T6 level, to locate from this reference the right transverse process of T9. With the probe placed longitudinally to visualize the needle in plane, we performed a craniocaudal puncture with a Perican® 18G 80 mm epidural needle. Upon contact with the transverse process, 1 mL of saline solution was injected, noting hydrodissection of the plane between the spinal erector muscle and the transverse process to ensure proper placement. Then, 15 mL of 0.5% bupivacaine was injected, which increased the width of the hydrodissection plane. A Perifix® catheter was then introduced to allow for continuous postoperative infusion of local anesthetics. The procedure described above was repeated on the left side to achieve bilateral analgesia and ensure that analgesia was achieved in the abdominal midline.

The OFA technique was performed according to our center's usual protocol. This protocol consists of intravenous preinduction with 0.5 mg/kg ketamine i.v., 1 mg/kg lidocaine i.v., 0.1 mg/kg dexamethasone i.v., 0.3 mcg/kg dexmedetomidine i.v., 250 mcg/kg esmolol i.v., and 2 g paracetamol i.v. Induction consisted of 2 mg/kg propofol i.v., 0.6 mg/kg rocuronium i.v., and 3 g magnesium sulfate i.v. Maintenance was achieved with 0.4–0.7 MAC desflurane to maintain a target bispectral index of 55, 0.2–1.5 mcg/kg/h dexmedetomidine i.v., 5–10 mcg/kg/min esmolol i.v., 10 mg/kg/h magnesium sulfate i.v., 1.5 mg/kg/h lidocaine, and 0.3 mg/kg/h ketamine i.v.

Intraoperative monitoring included electrocardiogram, SpO2 monitoring, noninvasive blood pressure (BP) assessments every 5 minutes, bispectral index (monitor integrated in the Primus® anesthesia station, Drägerwerk AG, Lübeck, Germany), and nociception level index (NOL index) assessments (PMD-200® monitor, Medasense Biometrics Ltd, Ramat Gan, Israel).

Preoperative pulmonary function tests showed a mild restrictive pattern; therefore, a ventilation strategy with low tidal volume and slightly elevated frequencies was planned for the maintenance of normocapnia or slight hypercapnia. In consideration of the patient's tendency to develop hypotension (median BP, 63–66 mmHg), a heart rate of <50 beats per minute, and an NOL index of <5, the dexmedetomidine dose was gradually reduced to 0.1 mcg/kg/h during the first hour of the intervention. Lidocaine, magnesium sulfate, ketamine, and esmolol doses were also gradually reduced until full suspension. The procedure continued exclusively with desflurane (for a bispectral index target of 50–55), 0.1 mcg/kg/h dexmedetomidine, and rocuronium as needed to maintain a clinically adequate neuromuscular block. The surgery lasted 4.5 hours, during which the patient did not develop any severe hemodynamic alterations that required the use of vasoconstrictors or inotropics. Furthermore, she did not require a fluid supply that was more than expected, given the wide peritoneal exposure. Twenty minutes before completion, 30 mg ketorolac i.v. and 8 mg ondansetron i.v. were administered, dexmedetomidine was discontinued, and 15 mL of 0.125% bupivacaine was administered in each ESP catheter. At the end of the intervention, the residual neuromuscular blockade was reversed with 200 mg sugammadex i.v. Finally, the patient was extubated when she reached an adequate spontaneous respiratory pattern.

During the postoperative period, we continued with the OFA protocol for 12 hours. This protocol consisted of 0.2 mcg/kg/h dexmedetomidine, 4 mg/kg/h magnesium sulfate, 1 mg/kg/h lidocaine, and 0.15 mg/kg/h ketamine. In addition, an infusion of 0.125% bupivacaine was maintained at 7 mL/h in each catheter and paracetamol (1 g i.v./8 h) and ketorolac (30 mg i.v./8 h) were prescribed as needed for pain control.

Four hours after surgery, the level of analgesia was examined via a prick test, which was started on each side through the center of the abdomen and moved proximally and caudally along the anterior surface of the body. The test revealed that analgesia was achieved from T3 to L2 on the right side (from the upper third of the thorax to the root of the thigh) and from T1 to L3 on the left side (from the ulnar area of the forearm and hand to the middle of the thigh), with extensive coverage of the central abdomen, which was the site of intervention.

During the first 48 hours after surgery, the patient consistently rated their pain on the numerical rating scale (NRS) as 0-1 out of 10 and did not require any additional analgesia. After 48 hours, both ESP catheters were removed and 1 g i.v./8 h paracetamol and 30 mg i.v./8 h ketorolac were administered as needed if the NRS score reached 1-2 out of 10.

Five days after surgery, the patient reported hypoalgesia at the level of the left thigh and decreased strength in the left leg. We consulted neurology to further examine the patient who also reported an isolated decreased strength in the quadriceps (5 right/2 left), without involvement of the psoas, biceps femoris, triceps sural, tibialis anterior, or tibialis posterior muscles. Muscle stretch reflexes remained alive except for an abolished left patellar reflex. We also noted hypoalgesia in territories scarcely congruent with the rest of the examination, at the level of the thigh in the L4 dermatome, and then at the level of the leg and foot in the L5 and S1 dermatomes. These findings were most consistent with a diagnosis of L4 neuropathy. These symptoms remitted within 48 hours and given that the patient had no other complications, she was discharged on the eighth postoperative day. Because blockade was established at the T9 level, away from the affected area, and the patient already had chronic pain problems and loss of strength in the lower limbs because of her underlying pathology, we did not consider it necessary to continue studying this event. In some publications, the realization of an ESP block has been related to the appearance of motor blockade or priapism [6, 7], possibly due to diffusion of the anesthetic to the anterior branches or the epidural space by the transforaminal route when a high volume of drug is administered [8–10]. However, in these cases, the complication appeared immediately after completion of the block and disappeared at a time consistent with its duration. In contrast, our patient developed the complication 5 days after the block and more than 72 hours after catheter removal. Excluding this neurological event, the postoperative period in this case was similar in quality and duration to that experienced by patients who undergo the most common anesthetic procedures performed at our center (general anesthesia and epidural analgesia).

## 3. Discussion

The erector spinae muscles comprise a series of muscle groups that extend along the cervical, thoracic, and lumbar regions. Theses muscles are located in the lateral groove of the spine and include the iliocostal, spinal, and long dorsal muscles. ESP block is a recently described technique [1], which is performed by injecting the anesthetic under the erector spinae muscles in the plane between them and the transverse processes of the underlying vertebrae. Diffusion of the local anesthetic into the paravertebral space through the spaces between adjacent vertebrae, and even to the epidural space, has been verified by magnetic resonance imaging and fluoroscopy with radiocontrast agent injection in previous studies [10, 11]. This diffusion allows the anesthetic to act on both the dorsal and ventral branches of the thoracic spinal nerves, as well as the communicating branches [1, 10–13]. Thus, ESP blocks often mimic the effects of retrolaminar or paravertebral blocks [13, 14]. Furthermore, since the erector spine muscles extend to the lumbar region, ESP blocks can produce abdominal analgesia if performed at the low thoracic or lumbar levels [2].

ESP block is an alternative to epidural or paravertebral blocks. Its safety profile is possibly better given an injection point far from the neuroaxis, pleura, and major vascular structures [15]. Furthermore, the transverse process, which represents the ultrasound target at any level, is easily visualized, and the introduction of the needle can be done in plane. This planar visualization of the needle and the target anatomical structures is an additional advantage in difficult cases [16], such as in patients with spinal deformities or spinal instrumentation [17]. Additionally, extensive craniocaudal diffusion of the anesthetic allows for wide coverage with a single injection [15]. In the case described here, the extent of anesthesia reached was greater than that described to date and that usually obtain in our clinical practice. We speculate that this was due to the patient's previous scoliosis surgery and consequent scar retraction, which may have reduced the elasticity of the fascial plane in which the local anesthetic is deposited.

The first publications on ESP block described thoracic-level treatment of neuropathic pain, rib fractures, thoracic vertebral surgery, and thoracic surgery [1, 14, 18–20]. Later, ESP block use was also reported in abdominal surgery for ventral hernia repair, bariatric surgery, nephrectomy, and hepatobiliary surgery [3, 12, 21–23].

Opioid-free analgesia (OFA) has evolved in the last two decades [24, 25], with the highest rates of use in patient groups with high rates of or higher risk of opioid side effects. These patient groups may include patients with morbid obesity, sleep apnea syndrome, history of hyperalgesia, complex pain, or fibromyalgia syndromes or patients with opioid addiction [4, 26–28]. However, OFA use beyond laparoscopic surgery remains limited in our practice. The combination of OFA and ESP blockade has been used successfully in extensive open surgery [17]. Here, we used this combination because of the large extent of the proposed surgical intervention, along with the patient's history of scoliosis surgery raised the possibility that an ESP block would not provide adequate analgesia. Furthermore, while the adverse effects of opiates previously noted by the patient were acceptable from an intraoperative management perspective, we did not require opioids in this case.

The multimodal anesthetic approach combining ESP block (conduction block) and opioid-free analgesia (nociception modulation) can eliminate the need for opioid analgesics during and after extensive abdominal surgery. Our findings open exciting new therapeutic possibilities. This potential is especially attractive for patients in whom epidural blockade and conventional opioid-based analgesia are contraindicated or show an unfavorable risk/benefit ratio.
